# Tailored Laser Structuring of Tungsten Carbide Cutting Tools for Improving Their Tribological Performance in Turning Aluminum Alloy Al6061 T6

**DOI:** 10.3390/ma16031205

**Published:** 2023-01-31

**Authors:** Robert Baumann, Yasmine Bouraoui, Uwe Teicher, Erik Selbmann, Steffen Ihlenfeldt, Andrés Fabián Lasagni

**Affiliations:** 1 Institute for Manufacturing Technology, Technische Universität Dresden, George-Baehr-Str. 3c, 01069 Dresden, Germany; 2 Fraunhofer Institute for Machine Tools and Forming Technology IWU, Nöthnitzer Straße 44, 01187 Dresden, Germany; 3 Institute of Mechatronic Engineering, Technische Universität Dresden, Helmholtzstraße 7a, 01069 Dresden, Germany; 4 Fraunhofer Institute for Material and Beam Technology IWS, Winterbergstraße 28, 01277 Dresden, Germany

**Keywords:** laser texturing, direct laser interference patterning, aluminum, tungsten carbide, turning

## Abstract

In times of societal development, sustainability has become a major concern for many manufacturers in the metal industries. In this context, surface texturing of cutting tools offers a promising approach in terms of reducing energy consumption and material waste. In this work, direct laser interference patterning is utilized for producing periodic line-like structures with spatial periods of 2.0 µm and 5.5 µm on rake-flank faces of cemented tungsten carbide cutting inserts. Structure depths up to 1.75 µm are reached by controlling the applied number of laser pulses. Turning experiments under lubricated conditions carried out on Al 6061 T6 parts with textured and untreated tools are performed to determine their tribological performances. The used textured cutting tools can effectively decrease machining forces up to 17% due to the corresponding improvement in frictional behavior at the tool/chip interface. Furthermore, the laser-processed tools produce thinner chips and decrease the surface roughness by 31% of the aluminum work piece.

## 1. Introduction

Excessive consumption of energy resources not only increases costs, but also has a hazardous impact on health and the environment due to its significant carbon footprint, greenhouse gas emissions, and release of toxic waste [1]. In this context, sustainable production involves a fine balance between improved productivity and minimizing the environmental impact [2,3,4]. During machining processes, such as metal cutting, high friction and heat are generated at the tool/chip interface. As a result, the tool experiences considerable wear, resulting in a shorter lifetime, and the quality of the newly machined surface is compromised [5]. Especially in the machining of aluminum alloys, this effect becomes relevant [6].

To overcome material losses caused by friction and wear, an excessive amount of cooling lubricants is used. This requires the disposal of metal cutting fluids containing machined particles, which are harmful for the environment. In addition, the use of large amounts of lubricants increases production costs [7,8]. Therefore, minimum quantity lubrication (MQL) which is based on the concept of near-dry machining has gained much wider acceptance in machining due to its effectiveness and environmental friendliness. However, MQL is typically associated with higher friction as well as a reduced tool life or requires the utilization of sophisticated cutting tools using, for instance, polycrystalline boron nitrite (PCBN) or polycrystalline diamond (PCD). Furthermore, dry cutting is not applicable for sticky materials such as titanium alloys and stainless steel [8,9].

To solve the challenges of dry and MQL cutting operations, surface texturing technologies can be implemented. Surface texturing usually refers to a defined modification of the surface topography by generating features with controlled shape and size [10,11]. In terms of the structuring of cutting tools, texturing techniques such as electric discharge machining (EDM), focused ion beam machining (FIB), and laser surface texturing (LST) have been already researched [12,13,14,15,16]. In general, it has been reported that textures can effectively improve the tribological performance of cutting tools, for example, reducing friction and wear or increasing their lifetime, since the structural elements act as debris entrapment as well as lubricant storage [11,12,13].

Among all these technologies, laser surface texturing has become a competitive technique due to its high accuracy, efficiency, attractive processing times, and eco-friendly nature [15]. Previous research reported a better cutting performance with nano-textured tools due to lower material accumulation in the structure grooves, in comparison to the micro-textured tools [17,18,19]. A promising approach for high-throughput laser texturing is the direct laser interference patterning (DLIP) technique. In DLIP, two or more laser beams are overlapped at the material surface for creating an interference pattern [17]. Depending on the number of laser beams as well as on the used geometrical configuration, different pattern geometries can be obtained, and the repetitive distance (period) can be reduced up to half of the laser wavelength. This technique has been already used for a wide range of applications, including wettability, anti-ice properties, and tribology [17,18,19,20]. In the case of tribological applications, several studies have demonstrated that periodic structures are more efficient in reducing friction coefficient and wear and increasing lubricant lifetime [21,22]. However, only a few investigations have been performed on hard metals [23]. Fang et al. implemented the DLIP technique using nanosecond pulses to manufacture line-like structures with a spatial period of ~12 µm on WC-CoNi samples with 14% cobalt content [22,23]. Tribological investigations were performed using a ball-on-disc tribometer under dry and lubricated conditions. It was reported that the line-like texture significantly decreased the friction coefficient under dry conditions. However, this effect was not observed under lubricated conditions. In this case, the pattern did not increase the hydrodynamic pressure required to build up a thick film [23].

Additionally, a comparative study on nanosecond and femtosecond fabricated DLIP textures on WC-CoNi was carried out to examine the treated surface integrity [24]. The measured spatial period on the samples treated with the ns-laser was 10.5 µm, and with the fs-laser, it was 3.46 µm. A cross-sectional inspection exhibited cracks and significant irregularities for the ns-textured surface, while for the fs-textured surface, these types of damages were absent. These damages were caused by higher heat-affect zone associated with the longer pulse durations of the ns-laser, which may affect the durability of the material during the machining processes [24]. Moreover, previous research reported a better cutting performance with nano-textured tools due to lower material accumulation in the structure grooves, in comparison to the micro-textured tools [25]. However, to the best of our knowledge, DLIP has not been applied so far for functionalizing cutting tools under machining conditions.

The aim of the present work is to improve the tribological performance of WC cutting tools as well as increase their lifetime using DLIP. Using this method, line-like structures are generated on the rake and flank face of cutting inserts coated with WC. Thereby, the effect of the created laser textures is evaluated depending on machining parameters such as cutting forces, chip formation, tool wear, and surface integrity of the machined workpiece.

## 2. Materials and Methods

### 2.1. Materials

For the laser structuring experiments, cemented tungsten carbide inserts (VBGW160400, HYPERION Materials and Technologies, Columbus, OH, USA) were used. The rhombic tools have a clearance angle α of 5° without chip breakers. Prior to the laser texturing, the samples were cleaned with ethanol. After the laser treatment, the tool noses were ground to obtain a nose radius r_ε_ of 0.4 mm with a resulting geometry of a VBGW160404. Following the laser process, the samples were stored under atmospheric conditions and without any other additional treatment. The material properties of the aluminum alloy EN-AW 6061 T6 are given in Table 1.

### 2.2. Laser Texturing Process

**Figure 1:**
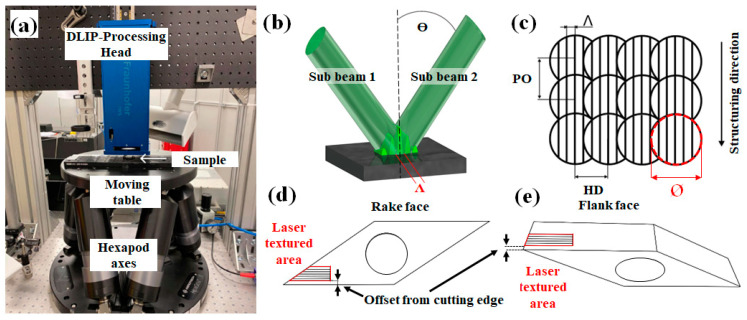
Experimental setup (**a**), schematic image of the intensity distribution and definition of the spatial period Λ (**b**), principle drawing of the texturing process strategy (**c**), top view of a schematic drawing of the rake face with the location of the laser processed area (**d**) and side view onto the flank face of a cutting tool with the schematic location of the laser processed zone (**e**).

The experimental setup of the equipment used for the laser experiments is illustrated in Figure 1a. The laser texturing process was carried out using a picosecond solid-state laser (Edgewave InnoSlab, PX200-3-GFH, Würselen, Germany) emitting radiation at a fundamental wavelength λ of 1064 nm and with a pulse width of 12 ps. This system is capable of delivering single pulses up to a repetition rate of 3 MHz, with a maximum average output power of 100 W (at 2 MHz). The Gaussian-shaped primary beam from the laser source is split into two sub-beams of identical intensity by a diffractive optical element (DOE). Subsequently, the sub-beams are parallelized through a prism and finally focused on the substrate surface using a focusing lens. The overlap of the sub-beams generates periodic line-like patterns with a spatial period Λ, as shown in Figure 1b. By controlling the prism position relative to the DOE, the incidence angle between the beams can be controlled, which allows adjustment of the spatial period Λ, according to Equation (1):

(1) $$\Lambda =\frac{\lambda }{2\sin\theta }$$

Within the presented study, two different spatial periods, 2.0 µm and 5.5 µm, were utilized. The overlap in the X direction (see Figure 1c), called hatch HD, was set to 10%, while the spots overlap in the Y direction, known as pulse to pulse overlap PO, was fixed at 90%. The HD depends on the spatial period Λ and the spot diameter Ø. The resulting interference lines are oriented parallel to the structuring direction. Aside from the processing parameters and the spatial period, the textures have been positioned at the WC-tools directly on the cutting edge or with an offset as shown in Figure 1d,e. Both the rake and flank faces of the cutting insert were treated by means of DLIP.

### 2.3. Mechanical Turning Experiments

**Figure 2:**
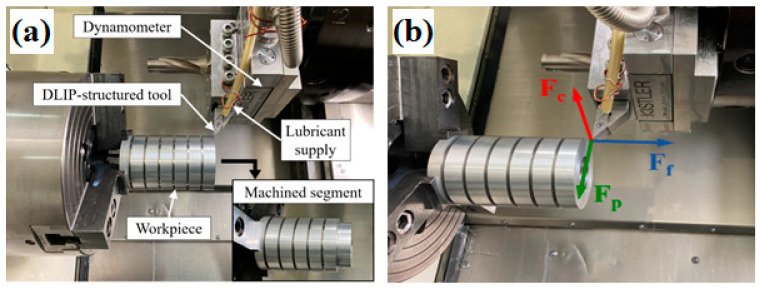
(**a**) Experimental design: Universal CNC lathe (type CTX alpha 300), dynamometer (type 9129AA) mounted on the tool holder, the cylindrical workpiece (Al 6061T6) preprocessed in defined segments; (**b**) Overview and direction of process forces (F_c_—cutting force, F_f_—feed force, F_p_—passive force).

The turning experiments were performed on aluminum EN-AW 6061 T6 cylindrical workpieces using a universal CNC lathe (DMG MORI—type CTX alpha 300, Bielefeld, Germany) equipped with a tool disc-type-turret, as shown in Figure 2a. The workpieces were preprocessed and subdivided into segments with a width of 12 mm. Each segment was used for the testing of one specific tool configuration. The tools were mounted on a Sandvik SVJBL 2020K16 tool holder (Sandvik Coromant, Sandviken, Sweden), which has a clearance angle α of 5°, a rake angle γ of 0°, and a cutting-edge angle β of 85°. The used cutting parameters were kept constant for all experiments, with a cutting depth a_p_ of 0.5 mm, a feed f of 0.15 mm/rev, and a cutting speed v_c_ of 500 m/min. The machining was carried out under flood-coolant conditions with the cooling lubricant CIMSTAR 501FF (CIMCOOL Fluid Technologies, Illnau-Effretikon, Switzerland), which was supported through a single lubricant nozzle (Figure 2).

Each segment of the workpieces was machined 10 times with thabove-mentioneded cutting depth (a_p_ = 0.5 mm) to achieve a defined cutting length l_c_ of 150 m. For statistic evaluation, the turning tests were repeated three times with a new, unused tool.

During the machining process, the cutting force components, more specifically the main cutting force F_c_, the feed force F_f_ and the passive force F_p_ (see Figure 2b), were recorded by a stationary piezoelectric 3-component dynamometer (Kistler, type: 9129AA, Winterthur, Switzerland) mounted directly on the tool holder, a charge amplifier (Kistler, type: 5007, Switzerland) and a Goldammer MultiChoice USB Basic (G0S-1034-4, Wolfsburg, Germany). Analysis of the data was done using National Instruments DIAdem-software (National Instruments, Austin, TX, USA). An overview of the used parameters of the laser textured tools is given in Table 2.

### 2.4. Surface Characterization

For measuring the surface topography of the laser-structured samples, confocal microscopic images (Sensofar S-Neox, Terrassa, Spain) were recorded. In addition, scanning electron microscopy (SEM) was employed (Zeiss Supra 40VP, Jena, Germany) for a more detailed analysis of the surface topography.

## 3. Results and Discussion

**Figure 3:**
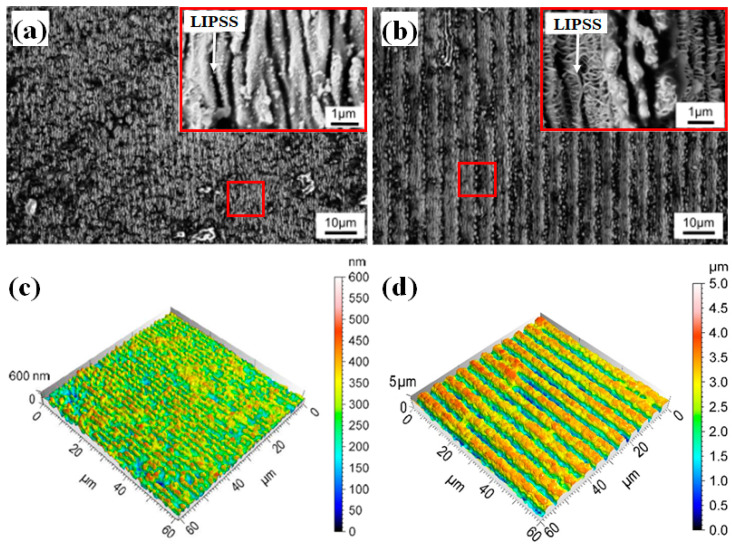
SEM images of DLIP textured cutting insert (Λ = 2.0 µm, NP = 40) (**a**), SEM images of DLIP textured cutting insert (Λ = 5.5 µm, NP = 60) (**b**), 3D-image with a magnification of 150X of DLIP-textured WC with Λ = 2.0 µm, NP = 40, Sd = 0.121 ± 0.014 µm, (**c**) and Λ = 5.5 µm, NP = 60, Sd = 1.21 ± 0.25 µm, (**d**).

In this work, the direct laser interference patterning DLIP technique was performed on cemented tungsten carbide turning insert samples with the aim of investigating the effects of the structure on the tribological performance of cutting tools. As a result, different surface topographies were produced with two different spatial periods of Λ of 2.0 µm, shown in Figure 3a,c and Λ of 5.5 µm, shown in Figure 3b,d.

It can be observed from the SEM images shown in Figure 3a,b that besides the line-like structure, low spatial frequency-LIPSS (LSFL) and high spatial frequency-LIPSS (HSFL) were generated. The spatial periods Λ of these nanostructures are in the range of ~500 nm and ~100 nm for LSFL and HSFL, respectively. Thus, the laser treatment of cemented tungsten carbide inserts results in the fabrication of hierarchical structures composed of linear periodic micro- and nano-patterns. The fabrication of the structures on the rake face and the flank face was carried out with identical processing parameters. Furthermore, the interference lines were oriented parallel to the cutting edge. In addition to the SEM images, confocal microscopy was applied for determining the achieved structure depth, shown in Figure 3c,d. As can be seen in Figure 3c, the spatial period Λ of 2.0 µm lead to shallow textures with structure depths Sd of 0.121 ± 0.01 µm and 0.162 ± 0.02 µm for applied 40 and 60 pulses, respectively. In contrast, the spatial period Λ of 5.5 µm with 40 pulses and 60 pulses achieved structure depth Sd of 1.35 ± 0.18 µm and 1.76 ± 0.32 µm, respectively. According to the manufacturer, the WC grain size of the used cutting insert ranges from 2 µm to 4 µm [26]. It was reported that the energy concentration increases proportionally with the grain size, as the coarse WC grains act as a barrier to energy dissipation due to their high thermal conductivity [27]. According to this, eruptions of the overheated material occurred, resulting in several structure damages for the smallest spatial period. However, for larger spatial periods Λ of 5.5 µm, this effect improved the formation of DLIP-textures. It can be concluded that the laser treatment for spatial period Λ of 2.0 µm produced nanostructures that consisted mainly of LIPSS rather than periodic patterns generated from the DLIP technique. Additionally, recoil drops can be observed on the structured surfaces (see Figure 3a,c) which confirms the eruption of overheated material and its redeposition.

**Figure 4:**
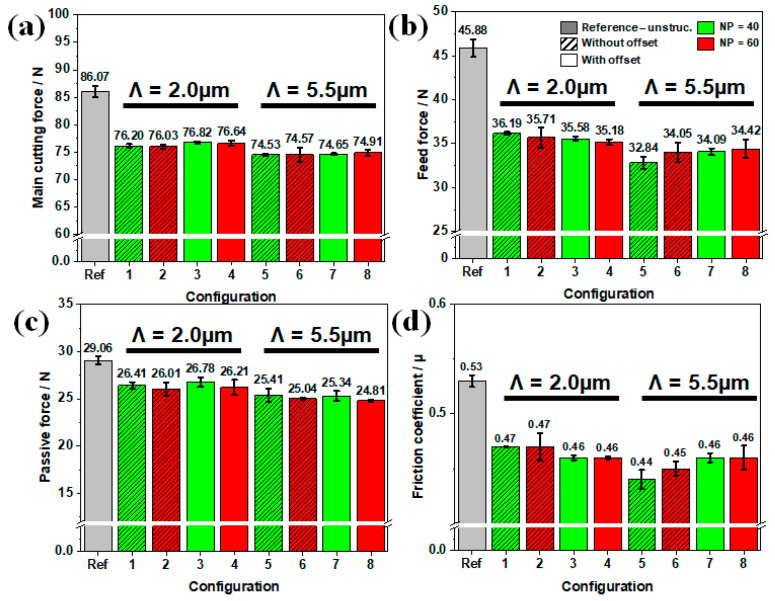
Machining forces evaluation for different configurations (conventional tool (Ref), (1–4) textured tools with Λ of 2 µm and (5–8) textured tools with Λ of 5.5 µm: (**a**) Main cutting force F_c_, (**b**) feed force F_f_, (**c**) passive force, and (**d**) the calculated friction coefficient µ.

The machining forces for the reference tool and the textured tools, which were recorded during the turning experiments are shown in Figure 4a. In comparison to the unstructured reference tool, it can be noticed that the machining forces significantly decreased for all textured tools.

In the case of the textured cutting tools with a spatial period Λ of 2.0 µm (see Figure 4a–d—configurations 1–4), the main cutting force F_c_ was reduced by 10.5–11%, the feed force F_f_ by 21–22%, and the passive force by 8–10%. While all configurations exhibited a similar lower main cutting force and passive force, configuration 4 (NP = 60, with offset) was the most effective in terms of feed force reduction. Moreover, the tools structured without an offset (configurations 1 and 2) showed slightly lower main cutting forces F_c_, compared to the tools with textures fabricated with an offset, shown in Figure 4a. In contrast, the lowest feed forces F_f_ were recorded for the tools having a texture with an offset (configurations 3 and 4).

The largest reduction of main cutting force was registered for the tools textured with a spatial period Λ of 5.5 µm (configurations 5–8) with a relative decrease of 12–13.5%, as shown in Figure 4a. Moreover, these configurations yielded a reduction of feed force F_f_ up to 26–28% and of a passive force of 12.5–14%, as illustrated in Figure 4b,c.

Additionally, configuration 5 (NP = 40; without offset from cutting edge) exhibited the best performances in terms of cutting force F_c_ and feed force F_f_ reductions. Nevertheless, in comparison with the other textured tool configurations with Λ of 5.5 µm, it exhibited the highest passive force. The textures manufactured with an offset from the cutting edge (configurations 7 and 8) resulted in slightly higher cutting forces F_c_ of 74.65 N and 74.91 N and feed forces F_f_ of 34.09 N and 34.42 N compared to the textures without an offset (configurations 5 and 6). It can be assumed that extending the texture to the cutting edge can further decrease the friction occurring at the tool/chip interface and thus decrease the machining forces.

In comparison with an untextured tool, the tool surface texturing by means of the DLIP technique effectively reduced the machining forces in all the studied configurations. It is well known that the friction occurring at the tool/chip interface strongly influences the machining force components [3]. Fabricated surface structures on the rake face reduce the real contact area. Consequently, the frictional behavior is improved, which in turn decreases the machining forces. Moreover, the generated structures act as microchannels, which can promote the lubricant penetration into the contact zone, supported by the capillary suction mechanism [28].

The coefficient of friction µ is a crucial parameter that determines the tribological conditions at the tool/chip interface. From the machining forces recorded during the turning experiments, the coefficient of friction can be calculated using the following equation [29]:

(2) $$µ=\frac{F_{c}\cdot \sin\gamma +F_{f}\cdot \cos\gamma }{F_{c}\cdot \cos\gamma -F_{f}\cdot \sin\gamma }$$

here *F_c_* is the main cutting force, *F_f_* is the feed force and *γ* is the rake angle (*γ* = 0°). The resulting coefficient of friction for all the configurations, and reference is shown in Figure 4d. In comparison with the unstructured reference tool, the friction coefficient decreased for all textured tools. The presence of micro textures on the tool rake face decreased the contact area at the tool/chip interface leading to lower friction. As expected, configuration 5 showed the best results in terms of friction reduction. This could be concluded from the low values of the feed force *F_f_* and main cutting force *F_c_*. Furthermore, the reduction in feed force *F_f_* by 28% was more evident compared to the other forces. As a result, configuration 5 exhibited the best frictional behavior due to correlation between feed forces and frictional forces for a zero-rake angle.

The chip compression ratio describes the ratio of the deformed to the undeformed chip thickness (*t*_2_/*t*_1_), indicating the degree of chip deformation. The segments of the formed chips with a length of 20 mm were weighted, and the average chip thickness *t*_2_ was determined. By assuming a rectangular cross section of the real chip, the deformed chip thickness *t*_2_ can be defined using the weight method of Equation (3) [30]:

(3) $$t_{2}=\frac{W}{\rho \cdot b_{1}\cdot l}$$

where *W* and *l* are the weight and the length of a piece of chip respectively, *ρ* is the material density, and *b*_1_ is the width of the uncut chip [5].

The undeformed chip thickness *t*_1_ is a constant value of 0.149 mm obtained from the feed rate and cutting-edge angle, according to Equation (4). In addition, for a zero-rake angle the shear angle Φ was calculated as follows [31]:

(4) $$\Phi =\arctan\left(\frac{t_{1}}{t_{2}}\right)$$

**Figure 5:**
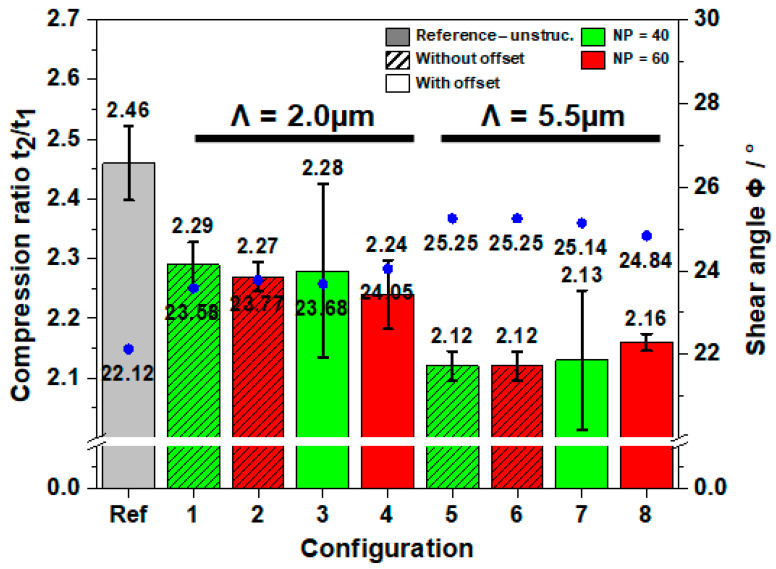
Calculated chip thickness ratios and shear ratio s for reference and textured tools.

The correlation between chip thickness ratios and shear angles for the reference tool and textured tools are illustrated in Figure 5. It was observed that all textured tools resulted in lower compression ratios, in comparison to the reference tool. This indicates that the textured tools could produce thinner chips, which reduce energy consumption and minimize the contact length and thus friction [32].

The values for the compression ratio obtained were consistent with the results of the friction coefficient discussed above. Moreover, it was noticed that textured tools with a larger spatial period of Λ = 5.5 µm (configurations 5–8) yielded smaller compression ratios, compared to the tool with smaller spatial periods of Λ = 2.0 µm (configurations 1–4). Among all configurations, the tool with configuration 5 exhibited the lowest compression ratio value, shown in Figure 5. This could be attributed to structure dimensions, which decreased the real contact area. However, the distance of the texture from cutting edge did not significantly influence the chip formation since the differences in chip thickness ratios are marginal.

A decrease in chip thickness ratio is associated with an increase in shear angle. This can be also observed with the results of the textured tools in Figure 5. Thus, tools texturing had an impact on chip forming mechanism. The chip formation implies shearing of the workpiece material along a plane from the cutting edge to the position at which the chip exits the workpiece [33]. The increase in shear angle for textured tools indicates that the shear plane decreased, and the cutting requires less shear force and thus the energy consumption is reduced. This is supported also by the observed lower cutting forces, which can be related to the reduced friction that facilitated the chip flow on the rake face. Interestingly, like the chip thickness ratio, the distance from the cutting edge did not contribute considerably to the chip formation. Conversely, the differences were found for a larger spatial period Λ of 5.5 µm. These observations resemble the results reported by other research groups, who state that the distance from the cutting edge is the most significant parameter in terms of the contact area but has minor impact to aluminum concentration on the tool surface [33].

For better understanding the wear mechanisms on the reference and the textured tools, scanning electron microscopy (SEM) analysis was performed at the rake and flank face for configuration 2 (Λ = 2 µm, NP = 60, without offset) and configuration 5 (Λ = 5.5 µm, NP = 40, without offset). These samples were selected as they showed the lowest cutting force for each spatial period.

**Figure 6:**
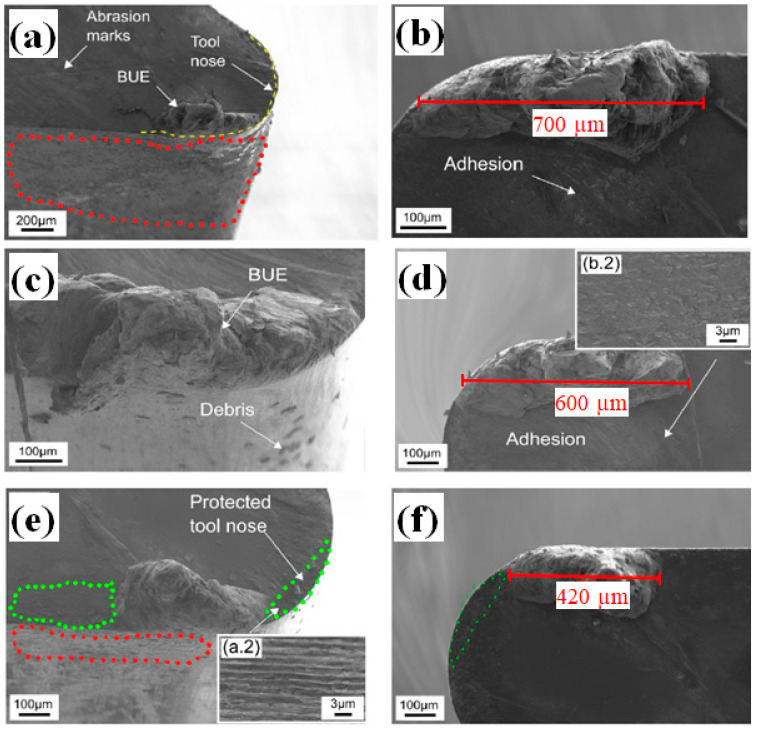
SEM images untextured tool tip for wear analysis: side view of rake-flank face at 25° tilting (**a**), enlarged image of rake face top view showing the BUE (**b**), textured tools with a spatial period of 2 µm: side view of tool tip and BUE formation of configuration 2 at 25° tilting (**c**), enlarged image of rake face top view showing the BUE for configuration 4 (**d**), textured tools with a spatial period of 5.5 µm: side view of tool tip and BUE formation of configuration 5 at 25° tilting (**e**), enlarged image of rake face top view showing the BUE for configuration 5 (**f**).

The SEM image of the reference tool tip, including flank and rake face after a cutting length of 150 m is shown in Figure 6a. It can be seen that serious abrasive wear and work material adhesion occurred over a large area on the flank face (area indicated in red), as well as on the rake face. An enlarged top view image of the tool nose is depicted in Figure 6b. Despite the relatively short cutting length, several ploughing marks resulting from tool and chip wear particles were noticeable, which represent the main factor of crater wear. Abrasive wear increases the real contact area and machining temperature at the tool/chip interface, resulting in work material welding onto the rake face. If the bonding between the chip material and the tool material is strong, random peeling of adhered material could remove tool material, causing severe adhesive wear [34]. Furthermore, the examination revealed the formation of build-up edge (BUE) over a length of 600 µm, which probably represents the cause of increased machining forces and friction. The formation of BUE could also affect the surface roughness of the machined workpiece. When machining aluminum alloys, severe adhesion occurs due the straight continuous chip formation, leading to significant adhesive wear and BUE formation.

SEM images of the tool tip with the configuration 2 with a spatial period of Λ = 2.0 µm is depicted Figure 6c,d. It can be seen that a large amount of work material adhesion occurred, forming a significant BUE. The length of the BUE was 700 µm, which is slightly higher compared to the reference tool. Furthermore, wear debris are clearly visible on the tool nose. However, the flank face did not exhibit important wear. It can be assumed that the textures on the flank face prevented the material adhesion, which reduced the adhesive and abrasive wear. Although, it can be noticed that structures on the flank face were completely suppressed after only l_c_ = 150 m cutting length. An explanation could be that the sliding movement of the tool against the workpiece flattened the texture by means of an abrasion mechanism. An SEM image of the tool rake face and the enlarged area, shown as an inset of texture can be seen in Figure 6d. When observing the tool nose, no cutting-edge chipping is apparent. Additionally, despite the BUE formation, it appears that the work material adhered slightly away from the cutting edge in some area of tool nose. This could be the reason why no breakage occurred. However, the inset of Figure 6e shows considerable chip material adhesion that almost completely covered the structures on the rake face. An enlarged magnification of the highlighted area clearly showed the presence of DLIP-textures, illustrated in Figure 6e. This indicates that the structure successfully served as wear debris entrapment, but the effect was limited due to the shallow depths. On the other hand, it seems that the existence of micro-textures at the cutting edge improved the build-up edge formation at this area.

Moreover, the texture acted as a trap for wear debris, preventing them from ploughing on the rake face. Thus, the frictional behavior was enhanced. However, this effect seems not to be present for a smaller period (Λ of 2.0 µm) due to greater surface irregularities that worsened the friction by acting as additional wear debris.

Finally, Figure 6e,f shows the tool tip with the large spatial period of Λ = 5.5 µm (configuration 5), featuring a significant reduction in work material adhesion. In fact, the textured area near the cutting edge and tool nose is clearly noticeable (areas indicated in green). In the inset of Figure 6d, the areas revealed that the structures were not completely covered with work material, which indicates that the benefits of tool texturing increased with an increase in structure dimensions, as presented in Figure 6e. Furthermore, it was observed that the material adhesion is less pronounced on the rake face at the area near the cutting edge and tool nose. It can be assumed that the availability of textures near the cutting edge shifted the chip flow on the rake face, preserving the tool nose from dramatic breakages. Although, BUE was also observed for this configuration, its length was 420 µm, considerably smaller compared to the reference tool and configuration 2. In the case of flank wear, minimal adhesive wear occurred near the cutting edge (area indicated in red). It is can be assumed that the textures acted as abrasive elements that removed the material from the workpiece. As a result, the removed material adhered to the flank face near the cutting edge. However, configuration 5 seems to reduce more effectively the wear occurrence due to improved lubrication conditions by the presence of textures at the cutting edge.

**Figure 7:**
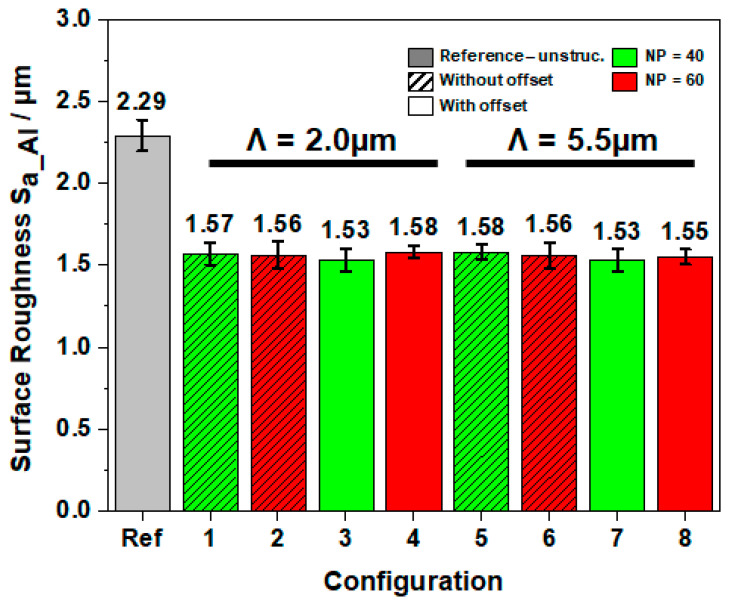
Measured micro-surface roughness after turning experiments of AL 6061 T6 for the different tool modifications.

The surface roughness is a crucial parameter that is closely associated to the machining conditions. In engineering applications, an improvement of product functional performance can be achieved by decreasing the surface roughness of the manufactured workpiece [35]. For the characterization of the quality of machined AL 6061 T6 workpiece with the developed tools, the micro-surface roughness S_(a_Al)_ was carried out using the confocal microscope Sensofar S Neox. The macro surface roughness resulting from the feed rate was not considered. Therefore, the measurements were performed on the three individually machined surfaces on three different positions each for statistical reasons. The resulting average surface roughness is depicted in Figure 7. In comparison with the unstructured reference tool, a significant decrease of 31% in average in surface roughness with the all textured tool was observed.

The tool surface texturing provided an enhanced lubrication at the contact area, and subsequently, reduced friction. In addition, the structures served as wear debris entrapment, reducing the ploughing effect. As a result, the tool nose integrity was preserved, which in turn increased the surface quality of the machined workpiece. However, configuration 5 did not show the lowest surface roughness, despite the best improvement in terms of machining forces, friction, and tool wear. Furthermore, the difference in resulting surface finish between the configurations remained marginal. It can be assumed that all texture types on the flank face affected the surface finish to the same extent. Even though configurations 7 and 8 (without offset) exhibited slightly lower values, this cannot be related to the distance of the structures from the cutting edge. In fact, configurations 3 and 4 were both manufactured with an offset but showed the lowest and highest values, respectively. It can be concluded that the absence of a clear trend was due to the random formation of build-up edge independently from the texture design. Further investigations regarding the relation between the build-up edge stabilization and surface finish, as well as residual stress must be carried out.

## 4. Conclusions

In this study, picosecond-laser-textured cemented tungsten carbide cutting tools were investigated regarding their tribological performances. Therefore, direct laser interference patterning (DLIP) with a two-beams configuration was used to fabricate line-like structures with spatial periods of Λ of 2.0 µm and Λ of 5.5 µm parallel to the cutting edge on rake and flank face of the inserts. By varying the process parameter, a maximal structure depth of S_d_ of 1.76 ± 0.32 µm was achieved. Furthermore, LSFL as well as HSFL could be observed with average spatial periods of approximately Λ of 500 nm and Λ of 100 nm, respectively. The following conclusions can be drawn:

- The turning investigations reveal that DLIP textured cutting tools improve the resulting surface roughness by 31% in comparison to an untextured tool. Furthermore, the main cutting forces were reduced up to 13.5%%, the feed forces by 28%, passive force by 14%, and friction coefficient by 28%;
- Moreover, the textured tools could produce thinner chips by 14% due to lower friction and improved lubrication at tool/chip interface. Thus, energy consumption could be reduced;
- The build-up edge formation was noticed for all textured tools and reference tool. However, structures with a larger spatial period were more effective in minimizing adhesive and abrasive wear, in comparison with smaller textures and reference tool.

In result, laser surface texturing by means of DLIP of rake-flank face of cutting tools reduced the real contact area, improved lubrication at chip/tool interface, and served as wear debris entrapment, thus reducing machining forces and friction. In the future, the mechanical cutting length will be prolonged for investigating tool wear as well as a restructuring process of the worn-off laser textured sections of the cutting insert. In future research, the combination of the DLIP technique with cryo-MQL could further improve the tool lifetime [35].

## Figures and Tables

**Table 1:** Material properties of EN-AW 6061 T6.

Parameters	Value
Mass density ρ/g/cm^3^	2.70
Young’s modulus E/MPa	70,000
Thermal expansion coefficient α/K^−1^ 10^−6^	23.0
Shear modulus G/GPa	26.3
Ultimate ensile strength R_m_/MPa	Min. 180
Tensile yield strength R_p0_._2_/MPa	Min. 110
Hardness Brinell/HBW	65

**Table 2:** Overview of all manufactured tool configurations for the turning experiments.

Parameter	Configuration Number							
	1	2	3	4	5	6	7	8
Λ/µm	2	2	2	2	5.5	5.5	5.5	5.5
Pulse numbers (NP)/-	40	60	40	60	40	60	40	60
Cutting offset/µm	0	0	200	200	0	0	200	200

## Data Availability

Not applicable.
